# Investigation on the Properties of Gneiss under Different Ground Stresses

**DOI:** 10.3390/s22041591

**Published:** 2022-02-18

**Authors:** Rui Wang, Yuncai Wang, Xianghui Deng, Yuan Qin, Bingxin Xie

**Affiliations:** 1 School of Civil and Architecture Engineering, Xi’an Technological University, Xi’an 710021, China; wangrui@xatu.edu.cn (R.W.); 15105879747@163.com (B.X.); 2 Xi’an Key Laboratory of Civil Engineering Testing and Destruction Analysis on Military-Civil Dual Use Technology, Xi’an 710021, China; 3 School of Water Resources and Hydropower, Xi’an University of Technology, Xi’an 710048, China; qinyuan@xaut.edu.cn

**Keywords:** gneiss, geostress, mechanical properties, failure mode

## Abstract

Initial geostress has great influence on the properties of gneiss. The physical and mechanical properties of gneiss vary considerably due to different initial geostresses, which exert a huge effect on the stability of underground engineering. In order to explore the influence of initial ground stress on the properties of gneiss. Changes in the physical properties (e.g., P-wave velocity and volumetric weight), mechanical properties (e.g., compressive strength, elastic modulus, and residual strength) and failure mode of gneiss are analyzed by conducting physical and mechanical tests on gneiss in different ground stress areas. The results show that high geostress can improve the pre-peak mechanical properties of gneiss, and weaken its post-peak mechanical properties. When the initial geostress is greater, the pre-peak mechanical properties are better, and the post-peak mechanical properties are worse. The failure mode of gneiss under high ground stress is primarily brittle failure. When the initial ground stress is greater, brittleness is stronger. According to the research results of this paper, it can provide the basis for the optimization and improvement of underground engineering support in gneiss strata with high geostress. The research results have important reference value and guiding significance for underground engineering construction in high geostress gneiss areas.

## 1. Introduction

With the rapid development of transportation infrastructure construction in recent years, the construction of underground engineering under complex geological conditions has become a development trend. Rock is a kind of discontinuous medium, so the physical and mechanical properties will change greatly under the influence of complex geological conditions, such as groundwater and high geostress [1,2,3]. If these changes are not considered in construction, the stability of underground engineering will be greatly affected [4,5,6].

Gneiss is a common rock in underground engineering construction, and scholars have conducted a series of studies on its physical and mechanical properties. Berčáková [7] explored the failure mechanisms of gneiss, and analyzed the influence of the weak surface on its mechanical properties and failure modes. Sammaljärvi [8] studied the microstructure of gneiss and explored the types of microscopic pores. Costa [9] explores the effect of temperature on the physical properties and microstructure of gneiss. It is found that the higher the temperature is, the more microscopic pores are, and the lower the P-wave velocity is. Mishra [10] explored the dynamic mechanical properties of gneiss and found that the peak strength of gneiss was related to strain rate, but dynamic modulus were not related to strain rate. Vettegren [11] explores the microscopic material composition on the failure surface of gneiss after shear failure, and studies the influence of mineral particles on rock failure. Trotta et al. [12] investigated the effect of the inhomogeneity of gneiss on mechanical properties. Yang et al. [13] conducted triaxial creep tests on gneiss under different freeze–thaw cycles, and explored the damage of freeze–thaw cycles on the properties of gneiss. Sun et al. [14] proposed the properties of gneiss based on variation in P-wave velocity. Zel et al. [15] studied the anisotropic characteristics of gneiss by using imaging methods, and examined the structure of gneiss through P-wave velocity and nuclear magnetic wave velocity. It can be seen from the above studies that scholars have studied the microstructure and mechanical properties of gneiss under different temperatures, freeze–thaw cycles, dynamic disturbances and other different states [16]. However, high geostress has a great influence on the mechanical properties of rocks. Scholars have less research on the mechanical properties of gneiss under high geostress, and have not yet explored the changes of gneiss under high geostress. The physical and mechanical properties of gneiss under high geostress are different from those under conventional geostress. Therefore, studying the influence of high ground stress on the mechanical properties of gneiss is highly significant.

With the deepening of rock mechanics research, scholars began to explore the influence of high ground stress on the mechanical properties. Feng [17] explored the deep fracture law of hard rocks under high ground stress, and observed that deep fracture exerted a significant effect on surrounding rocks. Pan [18] studied the change rule of coal oxidation heat release characteristics in a deep mine under high stress conditions. Zhu [19] explored the physical and mechanical properties of rock in high geostress areas, and proposed that large deformation under high ground stress is mainly caused by rock expansion effect. Liu [20] explored the fracture morphology caused by rock excavation under high geostress, and revealed the failure mechanism. Li et al. [21] performed triaxial unloading tests on coal rocks under high ground stress at different unloading rates. Zhang [22] explored the deformation characteristics of roadway surrounding rocks under high ground stress through theoretical analysis, field test, and laboratory test. The results showed that the strength and stiffness of the rock in a high ground stress were controlled by the local weakening area. Wang [23] proposed a supporting measure to prevent large deformation according to the failure mechanism of rock under high ground stress. Wang [24] explored the failure mechanism of soft rock under high geostress. Yang [25] explored the influence of ground stress at excavation failure zone. The aforementioned studies focused on rock mechanical properties under high ground stress and did not explore the influence of high ground stress. The variation of rock properties under different ground stresses is not explored, so the influence of high ground stress on the mechanical properties of rock cannot be obtained.

In summary, there are many studies on the mechanical properties of rock under high geostress, but there are few studies on the influence of high geostress on rock mechanical properties. In order to explore the influence of high ground stress on the mechanical properties of gneiss, the physical and mechanical tests of gneiss under different ground stress are carried out. The variation law of physical and mechanical properties of gneiss under different ground stress is summarized by analyzing the test results. Finally, the influence of high ground stress on the mechanical properties of gneiss is obtained.

## 2. Materials and Methods

### 2.1. Preparation of Specimens

One tunnel of the Baohan Expressway in Hanzhong City, Shaanxi Province is a typical deep-buried high ground stress tunnel. The surrounding rock grade in the tunnel site is Grade III, and the lithology is gneiss. To explore the influence of high ground stress on the mechanical properties of gneiss, high ground stress gneiss samples were collected from the deep-buried section of the tunnel with high in situ stress (YK183 + 681 to YK183 + 750). The sampling depth is about 950 m. Simultaneously, conventional in situ stress gneiss samples were collected in the shallow buried section of the tunnel entrance (YK189 + 161 to YK190 + 210). The sampling depth is about 150 m. In order to control the variables in the research process, the sampling depth of the same test block is controlled to be the same, and the sampling area is closer. In accordance with the requirements of the International Society for Rock Mechanics and the “Standard for Test Methods of Engineering Rock Mass” (GB/T50266-2013), two groups of rock specimens were used to make several cylindrical specimens with a diameter of 50 mm and a height of 100 mm.

**Figure 1:**
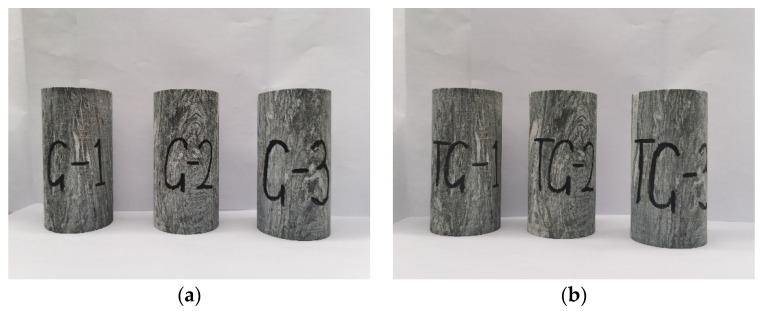
Gneiss specimens. (**a**) Gneiss specimens in the high geostress area for the uniaxial compression test. (**b**) Gneiss specimens in the high geostress area for the triaxial compression test.

The prepared test blocks were screened to avoid the influence of specimen defects on the test results. First, the surface without obvious cracks and broken test blocks was selected. Then, the size of the test blocks was checked and test blocks with a diameter and height error greater than 3 mm or that exhibited a non-parallelism of the end face greater than 0.05 mm were removed. Finally, the test blocks were numbered. For the zero confining pressure test of high geostress gneiss, the test blocks were numbered G-1, G-2, and G-3. For the different confining pressure test, the blocks were numbered TG-1, TG-2, TG-3, TG-4, and TG-5. For the corresponding conventional stress gneiss samples, the numbers were C-1, C-2, and C-3 and TC-1, TC-2, TC-3, TC-4, and TC-5. Some test blocks are shown in Figure 1.

### 2.2. Test Scheme

(1) Physical property tests

**Figure 2:**
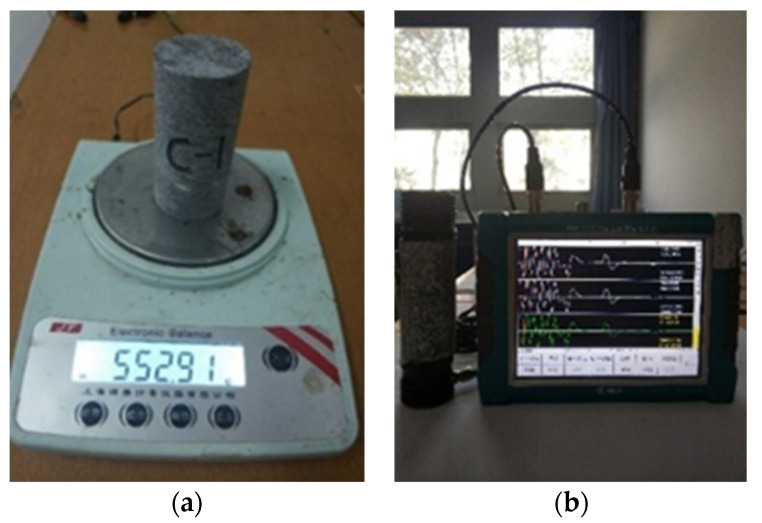
Major test steps. (**a**) Test block weighing. (**b**) Test block wave velocity test.

The diameter and height of each specimen were measured at room temperature by using a vernier caliper. Then, the mass of each test block was measured using an electronic balance, as shown in Figure 2a. Finally, an RSM-SY5 (T) acoustic wave tester manufactured by Wuhan Sinorock Technology Co., Ltd. (Wuhan City, Hubei Province, China) was used to test the longitudinal wave velocity of a specimen, as shown in Figure 2b.

(2) Uniaxial compression test

**Figure 3:**
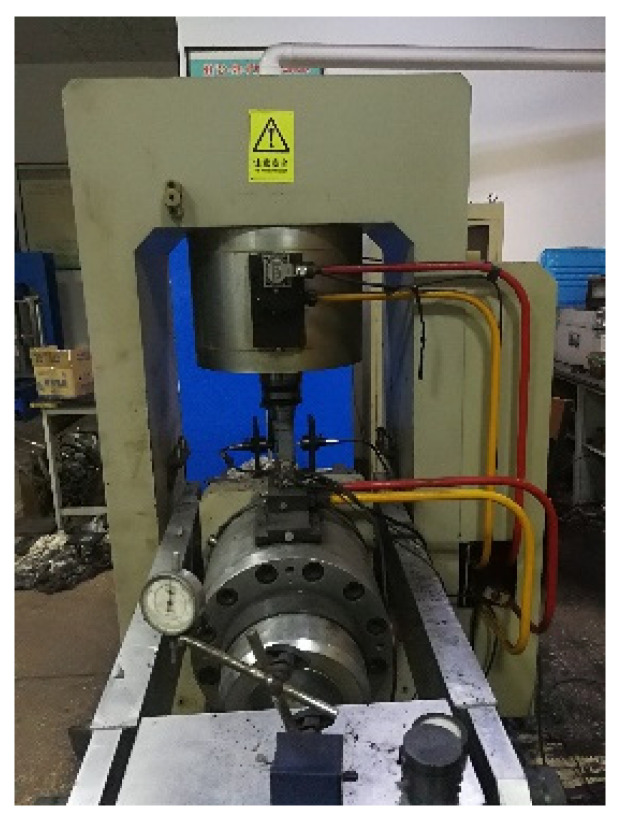
RMT-150 rock test system.

A uniaxial compression test was conducted using an RMT-150 rock test system. The test instrument is shown in Figure 3. An axial load was applied at a speed of 10 N/s until the specimen was destroyed. Longitudinal wave velocity was tested and recorded after the specimen was destroyed.

(3) Triaxial compression test

**Figure 4:**
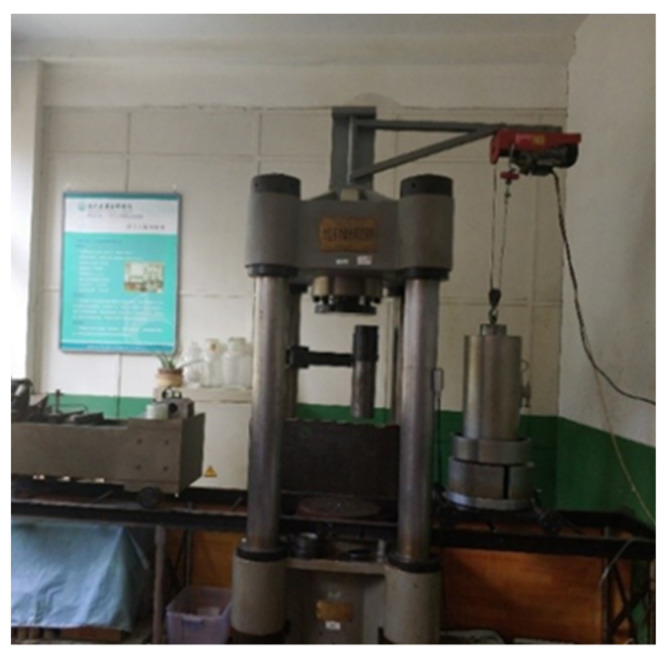
WDT-1500 large multifunctional material testing machine.

To explore the mechanical properties of gneiss further under different geostresses, triaxial compression tests were performed on the test blocks under different confining pressures. A triaxial compression test was conducted using a WDT-1500 large multifunctional material testing machine, as shown in Figure 4. The lateral pressure value was kept constant during the test until the specimen was destroyed. Then, the failure load value and failure condition were recorded. The confining pressure of the specimen was selected in accordance with the differential series: 5, 10, 15, 20, and 25 MPa.

## 3. Results and Discussion

### 3.1. Physical Property

The volumetric weight and initial wave velocity of gneiss in different stress areas are summarized in Table 1.

By analyzing the volumetric weight and initial wave velocity of gneiss with different ground stresses provided in Table 1, high ground stress is found to optimize the physical properties of gneiss compared with conventional ground stress, as follows:

(1) High geostress increases the volumetric weight of gneiss by 0.49% than that under conventional geostress. The volumetric weight of gneiss in the high geostress area is larger than that in the conventional geostress area, indicating that the internal structure of gneiss becomes closer under the action of high geostress.

(2) The average longitudinal wave velocity of gneiss in the high geostress area is 5.07 km/s, while that in the conventional geostress area is only 4.48 km/s. High geostress increases longitudinal wave velocity of gneiss by 13.17% than that under conventional geostress. The longitudinal wave velocity of gneiss in the high ground stress area is higher than that in the conventional ground stress area, indicating that the internal structure of gneiss is more compact under the action of high ground stress.

### 3.2. Uniaxial Compression Test Results

#### 3.2.1. Stress–Strain Curve

**Figure 5:**
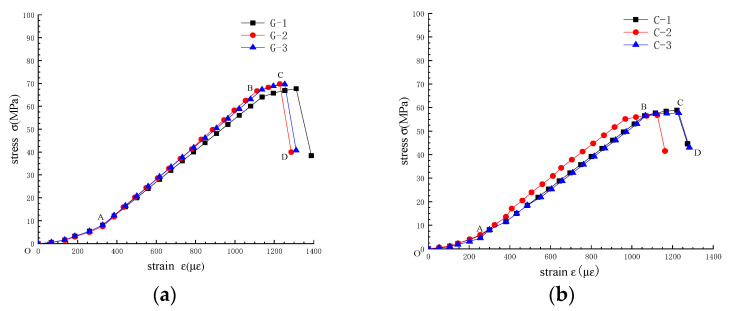
Stress–strain curves. (**a**) Gneiss in high geostress areas. (**b**) Gneiss in conventional geostress areas.

After organizing the data collected from the test, the stress–strain curve is shown in Figure 5.

As shown in Figure 5a,b, the change trend of the stress–strain curve of gneiss in different ground stress areas is basically the same and can be roughly divided into four stages: pore compaction stage (OA), elastic deformation stage (AB), plastic yield stage (BC), and plastic softening stage (CD). It can be seen from the BC and CD sections that when the load is greater than the yield strength, the gneiss will yield, and when the load is greater than the peak load, the gneiss will be destroyed rapidly. In the stress–strain curve, the slope of the curve represents the deformation modulus of the rock; that is, when the slope is greater, the deformation modulus is higher, and the capability to resist deformation is stronger. Figure 5 illustrates that the variation trend of gneiss is the same under different geostresses but the deformation modulus varies. Therefore, the strain generated during each stage of the stress–strain curve and the required stress values are summarized in Table 2.

Combining Figure 5 and Table 2, the deformation characteristics of gneiss at different in situ stress stages are analyzed as follows:

(1) The stress–strain curve of the pore compaction stage is an open upward parabola, indicating that the deformation modulus of gneiss is increasing during this stage. The primary reason is that gneiss contains minute initial pores, and these pores are gradually compacted under the action of external loads. During this stage, the deformation modulus of gneiss in the high geostress area is greater than that in the conventional geostress area, indicating that gneiss in the high geostress area exhibits stronger resistance to deformation.

(2) The stress–strain curve of the elastic deformation stage is approximately linear, and the slope of the curve remains basically unchanged, indicating that the capability of gneiss to resist deformation remains basically unchanged during this stage. The primary reason is that the initial pores in gneiss have been compacted, the interior is now dense, and no new cracks and damages occur. This gneiss can be regarded as an ideal elastomer. The deformation modulus of gneiss in the high geostress area is still greater than that in the conventional geostress area during this stage, indicating that the deformation resistance of gneiss in the high geostress area is still greater than that in the conventional geostress area.

(3) The stress–strain curve of the yield deformation stage is an open downward parabola, and the deformation modulus decreases continuously, indicating that the capability to resist deformation during this stage is decreasing. The primary reason is that the microelements in the rock begin to yield and produce plastic deformation after stress exceeds the plastic limit of gneiss. Although the capability of gneiss to resist deformation is continuously decreasing during this stage, the decline rate of the deformation modulus of gneiss in the high geostress area is significantly lower than that in the conventional geostress area. Moreover, the capability to resist deformation is still greater than that in the conventional geostress area.

(4) The stress–strain curve of the yield softening stage exhibits a downward oblique line, and stress during this stage decreases to the residual strength. The major reason is that the internal damage of gneiss accumulates at the local failure of the rock after reaching peak stress, and bearing capacity decreases. The stress loss of gneiss in the high geostress area is considerably larger than that in the conventional geostress area.

#### 3.2.2. Mechanical Properties

Physical and mechanical parameters, such as yield strength, peak strength, residual strength, elastic modulus, and longitudinal wave velocity, are counted and compared before and after the failure of gneiss in different geostress regions. The statistical results are provided in Table 3.

By analyzing the data in Table 3, the following conclusions can be obtained:

(1) The average yield strength of gneiss under high ground stress is 65.20 MPa, the standard deviation is 2.00, the average peak strength is 69.04 MPa, the standard deviation is 0.96, the average residual strength is 39.74 MPa, the standard deviation is 0.96. The average yield strength of gneiss is 56.08 MPa, the standard deviation is 0.66, the average peak strength is 57.77 MPa, the standard deviation is 0.85, the average residual strength is 43.08 MPa, and the standard deviation is 1.26. It can be seen from the above results that the standard deviation of test data obtained by gneiss specimens under different ground stresses is small, indicating that the data dispersion is small and the accuracy of the test results is high.

(2) The yield strength and peak strength of gneiss under high geostress increased by 30.32% and 19.51%, respectively, the residual strength decreased by 7.75%. The yield strength and peak strength of gneiss in the high geostress area are higher than those in the conventional geostress area, indicating that high geostress can improve the mechanical properties of gneiss before peak, increase the threshold for damage and failure in gneiss, and improve the strength of gneiss. Simultaneously, the residual strength of gneiss in the high ground stress area is less than that in the conventional ground stress area, showing that high ground stress makes the damage of gneiss more serious and residual strength after failure is lower. High geostress improves the pre-peak mechanical properties of gneiss but weakens its post-peak mechanical properties.

(3) The average elastic modulus of gneiss in the high geostress area is 73.72 GPa and that in the conventional geostress area is 66.90 GPa. The elastic modulus of gneiss in the high geostress area increases by 10%, indicating that high geostress improves the capability of gneiss to resist deformation.

(4) During the failure process of gneiss, its longitudinal wave velocity in the high ground stress area decreases by 44% and that in the conventional ground stress area decreases only by 30%, indicating that high ground stress weakens the post-peak mechanical properties of gneiss and makes the damage of gneiss more serious during the failure process.

(5) The larger the longitudinal wave velocity of gneiss, the smaller the internal damage is, the higher the peak strength is.

#### 3.2.3. Failure Mode

**Figure 6:**
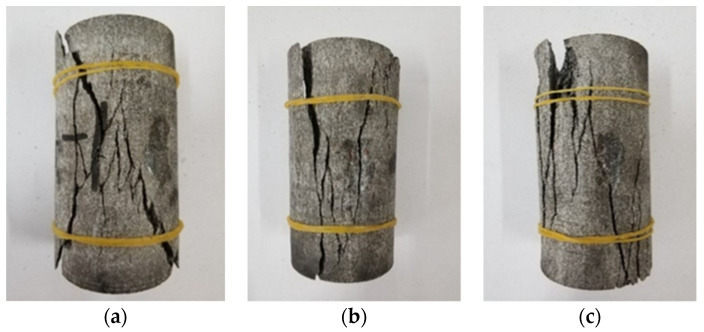
Specimen form of gneiss in the high geostress area. (**a**) G-1. (**b**) G-2. (**c**) G-3.

**Figure 7:**
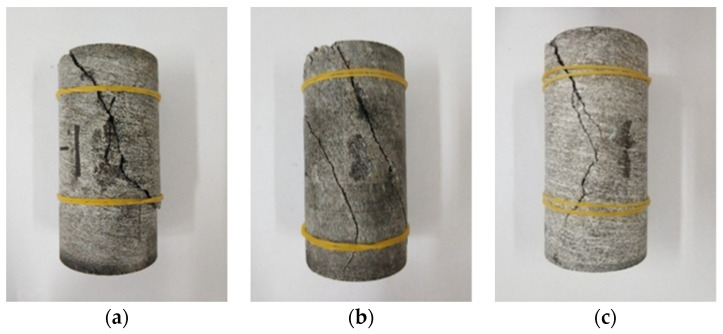
Failure mode of gneiss in the conventional geostress area. (**a**) C-1. (**b**) C-2. (**c**) C-3.

The failure mode of gneiss in different stress areas is analyzed in the uniaxial compression test, as shown in Figure 6 and Figure 7.

As depicted in Figure 6, the failure mode of gneiss in the high ground stress area is tension–shear composite failure. Many fracture surfaces are found after failure, the surface of a fracture surface is smooth, and the test block is broken after failure, producing more debris, which is typical of brittle failure. The main fracture surface forms an angle of approximately 30° with the loading direction of the specimen. Many derivative fracture surfaces around the main fracture surface occur throughout the entire specimen. The locations of derivative fracture surfaces are relatively random, and the angle is nearly parallel to the loading direction of the specimen largely because high ground stress produces more energy inside gneiss. During the failure process, energy is released, causing secondary damage to the rock, and thus, the rock is broken after failure.

As shown in Figure 7, the failure mode of gneiss in the conventional geostress area is shear failure, and one or two fracture surfaces are generated after failure. The surface of a fracture surface is rough, indicating plastic failure. After the test block is destroyed, the fracture surface forms an angle of approximately 20° with the loading direction. No derivative fracture surface is found around the main penetrating fracture surface largely due to gneiss having less internal energy in the conventional ground stress area. Thus, a derivative fracture surface is not produced after the destruction of only one fracture surface.

### 3.3. Triaxial Compression Test Results

#### 3.3.1. Stress–Strain Curve

**Figure 8:**
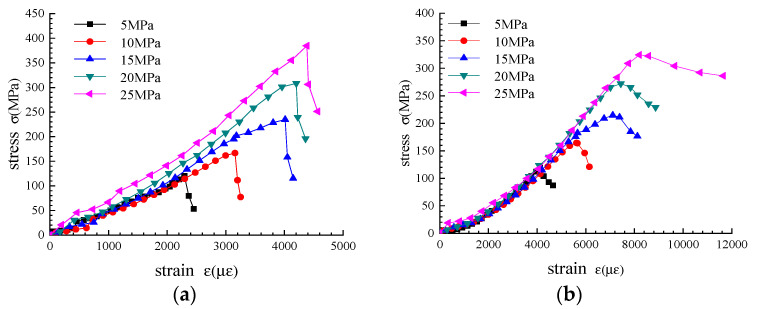
Stress–strain curves under different confining pressures. (**a**) Gneiss in high geostress areas. (**b**) Gneiss in conventional geostress areas.

The compression test results of gneiss under varying confining pressures in different ground stress areas are organized, and the stress–strain curves are drawn as shown in Figure 8.

As shown in Figure 8, the deformation law of gneiss in the triaxial compression test is basically the same as that in the uniaxial compression test. The stress and strain values at the end of each stage in the stress–strain curve are summarized in Table 4.

By analyzing the data in Table and Figure 8, the stress–strain variation law of gneiss under varying confining pressures in different in situ stress areas is obtained as follows:

(1) With the continuous increase in confining pressure, the yield strength, peak strength, and residual strength of gneiss under different geostresses are increasing; that is, the capability of gneiss to withstand load is increasing. Simultaneously, compared with the stress at the end of each stage before the peak stress under uniaxial compression, confining pressure significantly improves the bearing capacity of gneiss at each stage before the peak. When confining pressure is greater, bearing capacity increases more.

(2) With an increase in confining pressure, the stress loss of gneiss in the high geostress area increases continuously after reaching the peak stress, whereas the stress loss of gneiss in the conventional geostress area decreases continuously. High ground stress changes the mechanical properties of gneiss after peak, and residual stress decreases evidently. Simultaneously, when the confining pressure of high and low stress gneiss is greater, stress loss is also greater, indicating that when the initial geostress is greater, the post-peak stress of gneiss is reduced more.

(3) Under the same confining pressure, the deformation modulus of gneiss at each stage before reaching the peak stress in the high geostress area is greater than that in the conventional geostress area. When the confining pressure is greater, the gap between the two is larger. With an increase in confining pressure, the deformation modulus of gneiss in the high geostress area increases continuously at all stages before reaching the peak stress. Meanwhile, the deformation modulus of gneiss in the conventional geostress area does not change with an increase in confining pressure before reaching the peak stress. High geostress improves the capability of gneiss to resist deformation. With an increase in confining pressure, the deformation modulus of gneiss in the high geostress area will continue to increase at each stage before peak, indicating that when the initial geostress is greater, the capability of gneiss to resist deformation before peak is higher.

(4) Under the same confining pressure, the peak stress of gneiss in the high geostress area is greater than that in the conventional geostress area, but its residual strength is less than that of the latter. The difference in residual stress loss is larger with an increase in confining pressure. High ground stress weakens the post-peak bearing capacity of gneiss. When confining pressure is greater, stress damage after the peak stress is worse, indicating that when the initial ground stress is greater, the mechanical properties of gneiss are worse.

#### 3.3.2. Failure Mode Analysis

**Figure 9:**
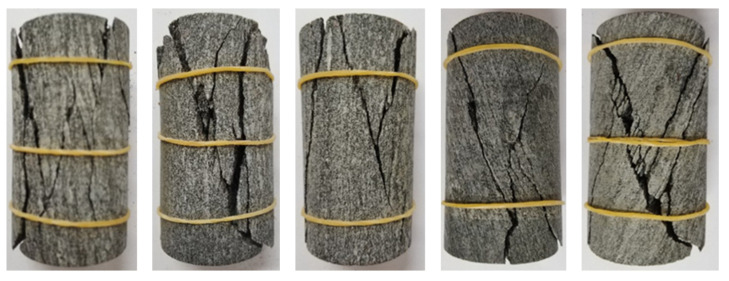
Failure modes of gneiss in the high geostress area. (**a**) 5 MPa. (**b**) 10 MPa. (**c**) 15 MPa. (**d**) 20 MPa. (**e**) 25 MPa.

**Figure 10:**
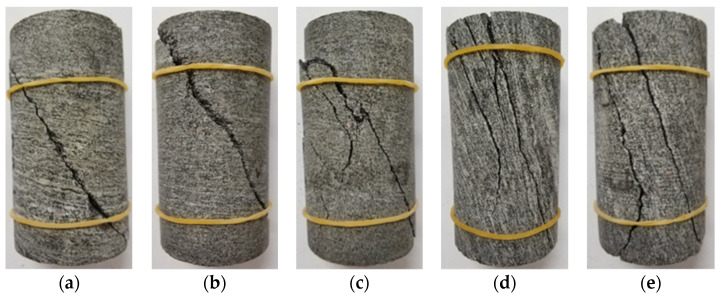
Failure modes of gneiss in the conventional geostress area. (**a**) 5 MPa. (**b**) 10 MPa. (**c**) 15 MPa. (**d**) 20 MPa. (**e**) 25 MPa.

The failure modes of gneiss in different geostress regions under varying confining pressures are presented in Figure 9 and Figure 10.

The analysis of Figure 9 shows that the failure mode of gneiss in the high geostress area is primarily tensile–shear composite failure. The fracture surface is smooth, exhibiting typical brittle failure. The angle between the main fracture surface and the loading direction is approximately 30°. With an increase in confining pressure, the direction of the main fracture surface remains basically unchanged, but the fracture surface is increased and its shape becomes more broken. The failure mode indicates that when confining pressure is greater, brittleness is stronger.

The analysis of Figure 10 shows that the failure mode of gneiss in the conventional geostress area is primarily shear failure. The fracture surface is relatively rough, which is indicative of plastic failure. The angle between the main fracture surface and the loading direction decreases with increasing confining pressure. The failure mode shows that plasticity is stronger with greater confining pressure.

### 3.4. Discussion

According to the mechanical properties and failure modes of gneiss under different ground stresses reflected by the test, the following reference can be provided for the design and construction of underground building structures in gneiss strata:

(1) It can be seen from the test results that high geostress can change the mechanical properties of gneiss, specifically, high geostress can improve the pre-peak mechanical properties of gneiss and weaken its post-peak mechanical properties. According to this result, it can provide a theoretical basis for the selection of excavation methods and the optimization of supporting parameters of building structures in gneiss strata under high geostress, and avoid the instability caused by ignoring the change of mechanical properties.

(2) High geostress can change the failure mode of gneiss and transform the failure mode from plastic failure to brittle failure. Therefore, the necessary early warning measures should be taken to prevent brittle failure and rockburst in the construction of underground buildings in gneiss strata under high geostress.

(3) When the gneiss under high ground stress is destroyed, the release of ground stress has secondary damage to the rock mass, resulting in more broken rock mass and lower longitudinal wave velocity. This feature can be used as a judgment basis for lithology and ground stress field in geological prediction.

## 4. Conclusions

The variation laws of the mechanical properties and failure modes of gneiss under different geostresses are explored by conducting physical and mechanical experiments on gneiss in high and conventional geostress areas. Through comparative analysis, the influences of high geostress on the mechanical properties and failure modes of gneiss are summarized. The specific conclusions are as follows:

(1) High ground stress makes gneiss denser, with less defects and better integrity. The longitudinal wave velocity and volumetric weight increased by 0.49% and 13.17%, respectively. Therefore, physical properties of gneiss under high geostress are better than those under conventional geostress.

(2) High geostress improves the pre-peak mechanical properties of gneiss. The yield strength and peak strength increased by 30.32% and 19.51% compared with the conventional stress. Various mechanical parameters, such as compressive strength and deformation modulus, are improved compared with those under conventional geostress. When the confining pressure of gneiss is greater in the high geostress area, mechanical properties are better. In the conventional geostress area, only the compressive strength of gneiss changes with confining pressure. The other mechanical properties do not change with confining pressure.

(3) High ground stress deteriorates the post-peak mechanical properties of gneiss. After reaching the peak stress, the stress loss of gneiss in the high geostress area is considerably larger than that in the conventional geostress area. Residual strength is considerably smaller than that in the conventional geostress area under the same confining pressure.

(4) High ground stress increases the brittleness of gneiss. After the failure of gneiss, its longitudinal wave velocity in the high geostress area decreases further, and failure mode is tension–shear composite failure. After failure, gneiss is more broken, and the fracture surface is smooth, indicating typical brittle failure. Brittleness becomes evident with an increase in confining pressure. However, integrity remains good after failure in the conventional in situ stress area, which is only a single or double section shear failure. The fracture surface is rough, indicating plastic failure. Plasticity is stronger when confining pressure is greater.

## Figures and Tables

**Table 1:** Statistical table of physical properties.

Specimen	Weight/g	Volume/cm^3^	Volumetric Weight/kN/m^3^	Initial Wave Velocity/km/s
G-1	552.02	197.23	27.34	5.08
G-2	557.65	198.81	27.44	5.08
G-3	552.81	197.42	27.44	5.05
C-1	552.91	198.01	27.34	4.57
C-2	555.40	199.40	27.24	4.54
C-3	555.56	199.40	27.24	4.33

**Table 2:** Strain value and stress at each stage.

Specimen	$\sigma_{c}$ (MPa)	$\varepsilon_{c}$ (με)	$\sigma_{e}$ (MPa)	$\varepsilon_{e}$ (με)	$\sigma_{p}$ (MPa)	$\varepsilon_{p}$ (με)	$\sigma_{r}$ (MPa)	$\varepsilon_{r}$ (με)
G-1	8.08	327.675	65.77	1195.05	67.69	1310.69	38.46	1387.82
G-2	7.52	327.752	62.51	1054.34	69.81	1227.82	40.00	1285.64
G-3	8.08	327.063	67.31	1137.26	69.62	1252.88	40.77	1310.72
C-1	5.77	255.63	56.54	1063.52	58.85	1220.96	44.62	1273.48
C-2	5.92	256.12	55.15	967.53	56.77	1125.06	41.54	1162.54
C-3	4.62	249.96	56.54	1066.49	57.69	1230.35	43.08	1282.51

Note: Subscript *c* in the table represents the value at the end of the compaction stage, *e* represents the value at the end of the elastic stage, *p* represents the value at the end of the yield stage, and *r* represents the value at the end of the residual strength stage. σ is the stress value and ε is the strain value.

**Table 3:** Statistical table of mechanical parameters.

Specimen	Yield Strength/MPa	Peak Strength/MPa	Residual Strength/MPa	Elastic Modulus/GPa	Initial Wave Velocity/km/s	Wave Velocity after Failure/km/s
G-1	65.77	67.69	38.46	72.10	5.08	2.84
G-2	62.51	69.81	40.00	75.91	5.08	2.83
G-3	67.31	69.62	40.77	73.15	5.05	2.82
SD	2.00	0.96	0.96	1.61	0.01	0.01
C-1	56.54	58.85	44.62	65.92	4.57	3.12
C-2	55.15	56.77	41.54	68.23	4.54	3.13
C-3	56.54	57.69	43.08	66.54	4.33	3.12
SD	0.66	0.85	1.26	0.98	0.11	0.01

Note: SD in Table 3 represents the standard deviation.

**Table 4:** Statistical table of mechanical parameters.

Specimen	$\sigma_{c}$ (MPa)	$\varepsilon_{c}$ (με)	$\sigma_{e}$ (MPa)	$\varepsilon_{e}$ (με)	$\sigma_{p}$ (MPa)	$\varepsilon_{p}$ (με)	$\sigma_{r}$ (MPa)	$\varepsilon_{r}$ (με)
TG-1	13.44	413.25	87.01	1855.25	120.33	2295.00	53.01	2454.00
TG-2	14.51	624.25	126.85	2479.01	166.53	3158.25	77.23	3253.01
TG-3	25.57	749.25	202.00	3179.75	234.82	4012.75	115.27	4150.06
TG-4	47.04	901.25	258.41	3472.75	308.17	4202.50	195.73	4360.50
TG-5	66.74	997.75	332.62	3852.75	384.78	4382.50	251.20	4566.76
TC-1	4.39	523.48	95.79	3685.65	119.23	4121.74	87.03	4656.52
TC-2	12.40	772.17	134.49	4743.91	164.30	5623.04	120.78	6150.43
TC-3	19.19	1303.04	182.42	5660.43	214.61	7109.13	176.47	8123.48
TC-4	27.78	1567.39	224.47	6170.02	271.84	7439.13	228.92	8868.26
TC-5	40.16	1735.22	263.95	6830.87	324.31	8203.04	286.16	11,652.17

Note: The parameters in Table 4 have the same meaning as those in Table 2.

## Data Availability

The raw/processed data required to reproduce these findings cannot be shared at this time as the data also form part of an ongoing study.

## References

[1] Gong F.Q., Si X.F., Li X.B., Wang S.Y. (2019). Experimental investigation of strain rockburst in circular caverns under deep three-dimensional high-stress conditions. Rock Mech. Rock Eng..

[2] Meng Y., Jing H., Zhou Z., Zhang L., Sun S. (2021). Experimental investigation on the mixed-mode fracture behavior of rock-like material with bedding plane. Theor. Appl. Fract. Mech..

[3] Zhang Y., Zhang X., Silva S.R.P., Ding B., Zhang P., Shao G. (2021). Lithium-Sulfur Batteries Meet Electrospinning: Recent Advances and the Key Parameters for High Gravimetric and Volume Energy Density. Adv. Sci..

[4] Deng X., Wang Y., Wang R., Xia D., Zhao Z. (2021). Application of modified Hoek–Brown strength criterion in water-rich soft rock tunnel. Geofluids.

[5] Xue Y., Liu J., Ranjith P.G., Zhang Z., Gao F., Wang S. (2022). Experimental investigation on the nonlinear characteristics of energy evolution and failure characteristics of coal under different gas pressures. Bull. Eng. Geol. Environ..

[6] Qian Q., Zhou X. (2018). Failure behaviors and rock deformation during excavation of underground cavern group for Jinping I Hydropower Station. Rock Mech. Rock Eng..

[7] Berčáková A., Melichar R., Souček K. (2019). Mechanical Properties and Failure Patterns of Migmatized Gneiss with Metamorphic Foliation Under UCS Test. Rock Mech. Rock Eng..

[8] Sammaljärvi J., Lindberg A., Voutilainen M., Ikonen J., Siitari-Kauppi M., Pitkänen P., Koskinen L. (2017). Multi-scale study of the mineral porosity of veined gneiss and pegmatitic granite from Olkiluoto, Western Finland. J. Radioanal. Nucl. Chem..

[9] Costa KO B., Xavier G.C., Marvila M.T., Alexandre J., Azevedo A.R.G., Monteiro S.N. (2021). Influence of high temperatures on physical properties and microstructure of gneiss. Bull. Eng. Geol. Environ..

[10] Mishra S., Khetwal A., Chakraborty T. (2018). Dynamic Characterisation of Gneiss. Rock Mech. Rock Eng..

[11] Vettegren V.I., Ponomarev A.V., Arora K., Mamalimov R.I., Shcherbakov I.P., Patonin A.V. (2017). Variation in the structure of the surface layer of a heterogeneous solid (gneiss) on a shear. Phys. Solid State.

[12] Trotta R.P., Barroso E.V., da Motta L.M.G. (2021). Migmatitic gneiss aggregates: Compositional, mechanical, and morphological responses to innate heterogeneity. Eng. Geol..

[13] Yang X., Jiang A., Zhang F. (2021). Research on creep characteristics and variable parameter-based creep damage constitutive model of gneiss subjected to freeze-thaw cycles. Environ. Earth Sci..

[14] Sun Q., Hu J., Zhang Y. (2021). Temperature and pressure dependence of P-wave velocity and electrical conductivity of gneiss: A review. Arab. J. Geosci..

[15] Zel I.Y., Petružálek M., Lokajíček T., Ivankina T.I., Kichanov S.E., Kozlenko D.P., Porosnicu I., Schnabl P., Pruner P., Duliu O.G. (2021). Assessment of structural, magnetic, and P-wave velocity anisotropy of two biotite gneisses from X-ray and neutron tomography. Tectonophysics.

[16] Liu J., Xue Y., Zhang Q., Wang H., Wang S. (2022). Coupled thermo-hydro-mechanical modelling for geothermal doublet system with 3D fractal fracture. Appl. Therm. Eng..

[17] Feng X.T., Pei S.F., Jiang Q., Zhou Y.Y., Li S.J., Yao Z.B. (2017). Deep fracturing of the hard rock surrounding a large underground cavern subjected to high geostress: In situ observation and mechanism analysis. Rock Mech. Rock Eng..

[18] Pan R., Gu Q., Chao J., Han X., Jia H., Wang J. (2021). Study on the spontaneous combustion oxidation properties of coal under the coupling effect of stress and temperature. Combust. Sci. Technol..

[19] Zhu Y., Chen L., Zhang H., Zhou Z., Chen S. (2019). Physical and Mechanical Characteristics of Soft Rock Tunnel and the Effect of Excavation on Supporting Structure. Appl. Sci..

[20] Liu G., Feng X.T., Jiang Q., Yao Z., Li S. (2017). In situ observation of spalling process of intact rock mass at large cavern excavation. Eng. Geol..

[21] Li X., Li H., Yang Z., Sun Z., Zhuang J., Song C., Wang X. (2021). Experimental study on triaxial unloading failure of deep composite coal-rock. Adv. Civ. Eng..

[22] Zhang S., Tang N. (2020). Local weakening laws and energy transformation of roadway surrounding rock with structural planes. Arab. J. Geosci..

[23] Wang Q., Jiang B., Pan R., Li S.C., He M.C., Sun H.B., Qin Q., Yu H.-C., Luan Y.-C. (2018). Failure mechanism of surrounding rock with high stress and confined concrete support system. Int. J. Rock Mech. Min. Sci..

[24] Wang Q., Pan R., Jiang B., Li S.C., He M.C., Sun H.B., Wang L., Qin Q., Yu H.-C., Luan Y.-C. (2017). Study on failure mechanism of roadway with soft rock in deep coal mine and confined concrete support system. Eng. Fail. Anal..

[25] Yang H.Q., Zeng Y.Y., Lan Y.F., Zhou X.P. (2014). Analysis of the excavation damaged zone around a tunnel accounting for geostress and unloading. Int. J. Rock Mech. Min. Sci..

